# Comparison between *in vitro* toxicities of tobacco- and menthol-flavored electronic cigarette liquids on human middle ear epithelial cells

**DOI:** 10.1038/s41598-020-59290-y

**Published:** 2020-02-13

**Authors:** Yoon Young Go, Ji Yoen Mun, Sung-Won Chae, Jiwon Chang, Jae-Jun Song

**Affiliations:** 1 Department of Otorhinolaryngology-Head and Neck Surgery, Korea University College of Medicine, Seoul, Korea; 2 Department of Otorhinolaryngology-Head and Neck Surgery, Hallym University College of Medicine, Seoul, Korea

**Keywords:** Autophagy, Medical research

## Abstract

Since electronic-cigarettes (e-cigarettes) are considered less toxic than conventional tobacco smoking, the use of e-cigarettes has increased, and the market for e-cigarette liquids (e-liquids) is continuously increasing. However, many studies showed that e-cigarettes may cause various harmful effects in lung, oral and heart. In this study, we investigated the effects of e-liquids on otitis media (OM) using human middle ear epithelial cells (HMEECs). Menthol-flavored e-liquid induced significant cell death in HMEECs (IC_50_: 1.45 ± 0.14%) and tobacco-flavored e-liquid led to increase in inflammatory cytokine levels and higher mucin production. Flavored e-liquids decreased the mRNA levels of genes encoding epithelial sodium channels (ENaCs) in HMEECs. Apoptosis and autophagy reactions were induced by exposure of HMEECs to menthol- and tobacco-flavored e-liquids. Tobacco-flavored e-liquids caused a greater increase in the levels of autophagosome marker, LC3-II, compared to menthol-flavored e-liquids, which was followed by cell death. These results demonstrate that flavored e-liquids cause cytotoxicity via apoptosis, autophagy, inflammatory response, and mucin production in HMEECs. The flavors present in e-liquids might be a risk factor for the development of otitis media.

## Introduction

Otitis media (OM) is an ear infectious disease and its symptoms include pain, inflammation, and flow of fluid out of the middle ear cavity. Particularly, almost 50% of children experience OM under the age of 3 years^1^. The middle ear infection usually presents as acute otitis media (AOM) within one week, but can develop into chronic otitis media (COM). COM persists over 2 weeks, and can cause serious inflammation in the inner ear and hearing loss. OM is typically induced by bacterial and viral infection in the middle ear during the dysfunction of the eustachian tube and nasal cavity^2^. However, recent studies have investigated the relationship between environmental factors (smoke, diesel particles, urban particles, and Asian sand dust) and OM incidence^3–6^.

Electronic cigarettes (e-cigarettes) are available on the market as an alternative to conventional tobacco smoking. E-cigarettes were introduced to help reduce smoking habit by providing nicotine selectively from 0 to 36 mg/mL^7^ in various flavors of e-cigarette liquids (e-liquids). Many users consider e-cigarettes to be safer than tobacco cigarettes, which led to increase in marketing of e-cigarettes for nicotine delivery. However, several animal and human cohort studies have been conducted since 2014 related to the harmful effects of e-cigarettes on human health, including increased tumorigenicity in lung and bladder^8^, cardiovascular system^9^, and alterations in the oral and gut microbiota in humans^10^.

E-cigarettes vaporize flavor and nicotine-containing fluids through a heating coil in an atomizer, and the user inhales the aerosol. Although e-liquids do not contain harmful agents, such as tar, benzene, carbon monoxide, and formaldehyde, their safety concerns have been raised, except for nicotine addiction^11^. The e-liquid simply consists of propylene glycol (PG), vegetable glycerol (VG), various flavors, and nicotine. However, many recent studies have reported that flavorings in e-cigarettes induce toxicity in the human lung and airway epithelial cells^12–15^. Gurjot *et al*. determined the mechanisms of toxicity of flavoring agents, which may exert their effects by increasing oxidative stress, cytotoxic responses, immune-mediated responses, and DNA damage^11^.

In our previous report, we showed the cytotoxicity of e-liquids on OM, using human middle ear epithelial cells (HMEEC) as an *in vitro* model^16^. However, the potential mechanism of cytotoxicity is still unclear and comparative studies on the effects of different flavor-containing e-liquids on HMEECs have not been conducted. Since flavoring agents are well-known causes of cytotoxic responses and inflammation in cells^11^, flavoring agents in e-cigarettes may be associated with the development of OM. Therefore, the purpose of this study was to evaluate the effects of OM, including inflammatory response, mucin production, dysregulation of water channels, and cytotoxic effects on HMEECs by treatment with two different flavors of e-liquids.

## Results

### E-liquids reduced viability of HMEECs in a dose-dependent manner

In our previous study, 73 bottles of flavor-containing e-liquids from 12 different brands were analyzed in terms of cytotoxicity on HMEECs. These results showed that exposure to e-liquid reduced HMEEC viability, regardless of the e-liquid brand and flavor. Among the flavors, menthol-flavored e-liquids, considered to be the most toxic e-liquids, significantly decreased the viability of HMEECs (average IC_50_ = 1.85 ± 0.80%, n = 28). Tobacco-flavored e-liquids exhibited moderate cytotoxicity (average IC_50_ = 3.36 ± 0.13%, n = 13) in all solutions and were considered as a control for flavored e-liquids^16^.

**Figure 1:**
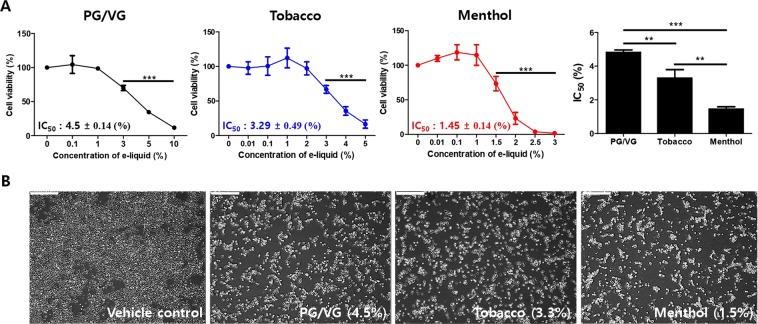
E-liquid reduced cell viability of HMEECs in a dose-dependent manner. (**A**) HMEECs were treated with PG/VG, tobacco- and menthol-flavored e-liquids for 24 h in a various concentration of e-liquid (0.01 to 10%). The control group was not exposed to e-liquids. The cellular cytotoxicity was determined by cell counting assay in HMEECs. Cell viability was reduced by exposure to e-liquids in a dose-dependent manner. The multiple independent experiments were performed (*n* = 6) and the average IC_50_ (half maximal inhibitory concentration) values were calculated using the ED_50_ plus v 1.0 software and showed as mean ± SD (PG/VG: 4.5 ± 0.14, tobacco-flavored: 3.29 ± 0.49, and menthol-flavored: 1.45 ± 0.14). **p < 0.01 and ***p < 0.001 compared to the corresponding control. (**B**) HMEEC morphology after exposure to the average IC_50_ values of each e-liquid for 24 h (PG/VG: 4.5%, Tobacco: 3.3%, and Menthol: 1.5%). Vehicle control is untreated HMEECs. E-liquids-treated groups showed 50% cell death compared to the control group.

To investigate the cytotoxicity of these two differently flavored e-liquids on HMEECs, we first performed a cell viability assay, and then, compared the results with IC_50_ values. PG/VG was used as a solvent control and a PG:VG of 5:5 was used because the composition of PG and VG vary in e-liquids. The cell viability was remarkably decreased in time- and concentration-dependent manner in cells exposed to both flavored e-liquids (Fig. S1 and Fig. 1A). The IC_50_ value of PG/VG was 4.5 ± 0.14%, menthol-flavored e-liquids was 1.45 ± 0.14%, and tobacco-flavored e-liquids was 3.29 ± 0.49% for HMEECs after treatment for 24 h, indicating that the cytotoxicity of menthol-flavored e-liquid was significantly higher than that of tobacco-flavored e-liquids in our results (Fig. 1A). Representative images of cytotoxicity of e-liquids are shown in Fig. 1B. Microscopic evaluation showed that the number of viable cells decreased by approximately 50% in PG/VG and e-liquid-treated groups, but not in untreated control cells.

### E-liquids induced the expression of inflammatory cytokines in HMEECs

**Figure 2:**
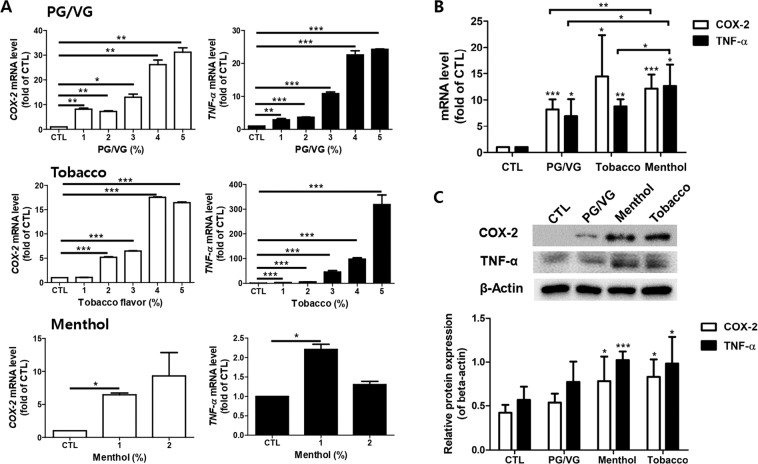
E-liquids stimulated the expression of inflammatory cytokines in HMEECs. (**A**) HMEECs were treated with PG/VG (1 to 5%), tobacco-flavored e-liquid (1 to 5%), and menthol-flavored e-liquid (1 and 2%) for 24 h. Quantitative real-time PCR was performed to evaluate inflammatory cytokine gene such as *COX-2* and *TNF-α* expression levels. The expression levels of *COX-2* and *TNF-α* significantly increased in e-liquid-concentration-dependent manner. (**B**) Cells were exposed to the average IC_50_ values of each e-liquid for 24 h (PG/VG: 4.5%, Tobacco: 3.3%, and Menthol: 1.5%). Both flavored e-liquids significantly increased the mRNA levels of *COX-2* and *TNF-α* on HMEECs. mRNA expression of *COX-2* and *TNF-α* was significantly increased in menthol-flavored e-liquid-treated group compared to in PG/VG group. (**C**) Cells were treated with PG/VG (4.5%), tobacco-flavored e-liquid (3.3%), and menthol-flavored e-liquid (1.5%) for 24 h. The results showed that the levels of COX-2 and TNF-α protein levels were increased by treatment with e-liquids. Densitometric analysis of western blot is shown as a graph. Similar to their mRNA profiles, COX-2 and TNF- α protein expression was significantly higher following tobacco- and menthol-flavored e-liquid exposure compared to the control group. Western blot data were quantified and normalized to β-actin levels. All data were obtained from three independent experiments and the error bars indicate the mean ± SD. *p < 0.05, **p < 0.01, and ***p < 0.001 compared to the corresponding control.

It has been known that high COX-2 and TNF- α expression contribute to the development of OM^13,17^. The mRNA levels of *COX-2* and *TNF-α* were analyzed to determine the inflammatory response of HMEECs following treatment with e-liquids. The expression levels of *COX-2* and *TNF-α* were significantly increased in a dose-dependent manner in HMEECs cultured with the PG/VG, menthol-flavored, and tobacco-flavored e-liquids compared to that in the untreated control cells (Fig. 2A). In addition, treatment of HMEECs with each of the e-liquid increased the mRNA levels of *COX-2* and *TNF-α*, as shown in Fig. 2B. Exposure to the menthol-flavored e-liquids on HMEECs was significantly evaluated the expression levels of *COX-2* and *TNF-α* gene compared with PG/VG treatment. Western blot analysis also showed significantly upregulated COX-2 and TNF-α protein levels when the cells were exposed to flavored e-cigarette liquids (Fig. 2C). These results suggested that the expression levels of inflammatory cytokines, such as COX-2 and TNF-α, were upregulated when HMEECs were exposed to e-liquids.

### E-liquids increased mucin production in HMEECs

**Figure 3:**
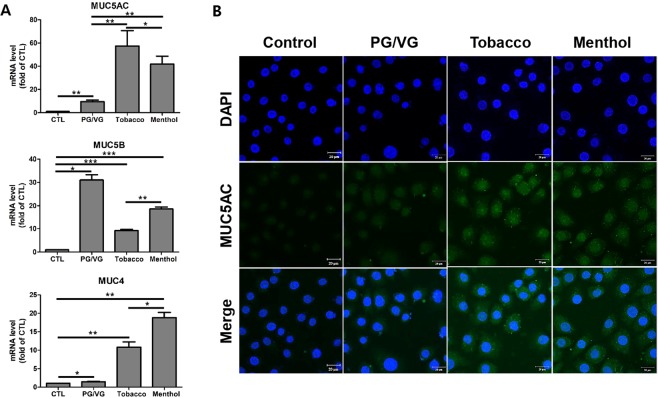
E-liquids increased mucin production in HMEECs. (**A**) HMEECs were exposed to e-liquids at the IC_50_ concentration for 24 h (PG/VG: 4.5%, Tobacco: 3.3%, and Menthol: 1.5%), and quantitative RT-PCR was performed to investigate the expression levels of mucin genes such as *MUC4*, *MUC5AC*, and *MUC5B*. Tobacco- and menthol-flavored e-liquids significantly increased the mRNA expressions of *MUC4* and *MUC5AC*. Data were obtained from three independent experiments and the error bars indicate the mean ± SD. *p < 0.05, **p < 0.01, and ***p < 0.001 compared to in the corresponding control. (**B**) Cells were treated with e-liquids at the IC_50_ concentration for 24 h (PG/VG: 4.5%, Tobacco: 3.3%, and Menthol: 1.5%). Confocal microscopic images showing MUC5AC expression (green) in HMEECs as detected by immunofluorescence staining. MUC5AC expression in cells was higher in e-liquid-treated groups than in the control group. Nuclei were counterstained with DAPI (blue).

Mucins are classified with the 19 families of glycoproteins, produced by epithelial tissue in animals for protection against pathogens and particulate matter as part of the immune function. *MUC1*, *MUC4*, *MUC5AC*, and *MUC5B* are known to be expressed in human epithelial cells^18,19^, which is highly related to the pathophysiology of OM^19^. In this study, we examined mucin production in HMEECs stimulated with e-liquids. The mRNA levels of *MUC5AC* and *MUC4* were significantly higher in cells treated with flavored e-liquids compared to those in control and PG/VG groups, showing increases of up to 6- and 20-fold, respectively (Fig. 3A). Interestingly, treatment with PG/VG markedly increased the mRNA levels of *MUC5B*. MUC5AC expression in HMEECs was evaluated by immunofluorescence staining and the results indicated that MUC5AC expression was significantly elevated by tobacco-and menthol-flavored e-liquid (Fig. 3B). These results showed that e-liquids induced mucin production in HMEECs.

### E-liquids caused dysregulation of water channels in HMEECs

**Figure 4:**
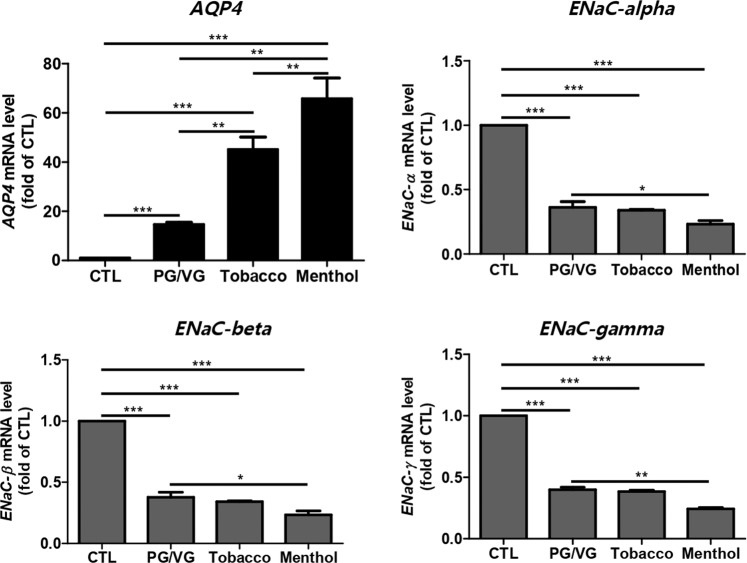
E-liquids caused dysregulation of water channels in HMEECs. HMEECs were treated with PG/VG and flavored e-liquids at the IC_50_ concentration for 24 h (PG/VG: 4.5%, Tobacco: 3.3%, and Menthol: 1.5%). Untreated cells were used as a control. Expression levels of genes related to water channels, such as *AQP4* and *ENaC* family members, were specifically investigated as otitis media markers by quantitative real-time PCR. Tobacco- and menthol-flavored e-liquids significantly induced an increase in *AQP4* expression and decrease in ENaC family gene expression compared to the control and PG/VG groups. All data were obtained from three independent experiments and the error bars indicate the mean ± SD. *p < 0.05, **p < 0.01, and ***p < 0.001 compared to the corresponding control.

Patients with OM show symptoms such as mucosa membrane expansion and fluid accumulation in the middle ear^20^. These symptoms are induced by dysfunction of the water transport system, such as dysregulation of the middle ear epithelial sodium channel (ENaC) or aquaporin (AQP) water channel^20,21^. In our study, the expression level of the *AQP4* gene was remarkably increased in HMEECs exposed to e-liquids compared to that in the untreated group. The expression levels of the *AQP4* gene were increased by up to 20-, 40-, and 60-fold in HMEECs exposed to PG/VG, tobacco-flavored e-liquid, and menthol-flavored e-liquid, respectively. Although the gene expressions of ENaC family members, such as *ENaC-α*, *ENaC-β*, and *ENaC-γ*, were decreased by both flavored and non-flavored e-liquid solutions, treatment with menthol-flavored e-liquid significantly downregulated the mRNA levels of ENaC family members in HMEECs (Fig. 4).

### E-liquids induced apoptosis and autophagy signaling in HMEECs

**Figure 5:**
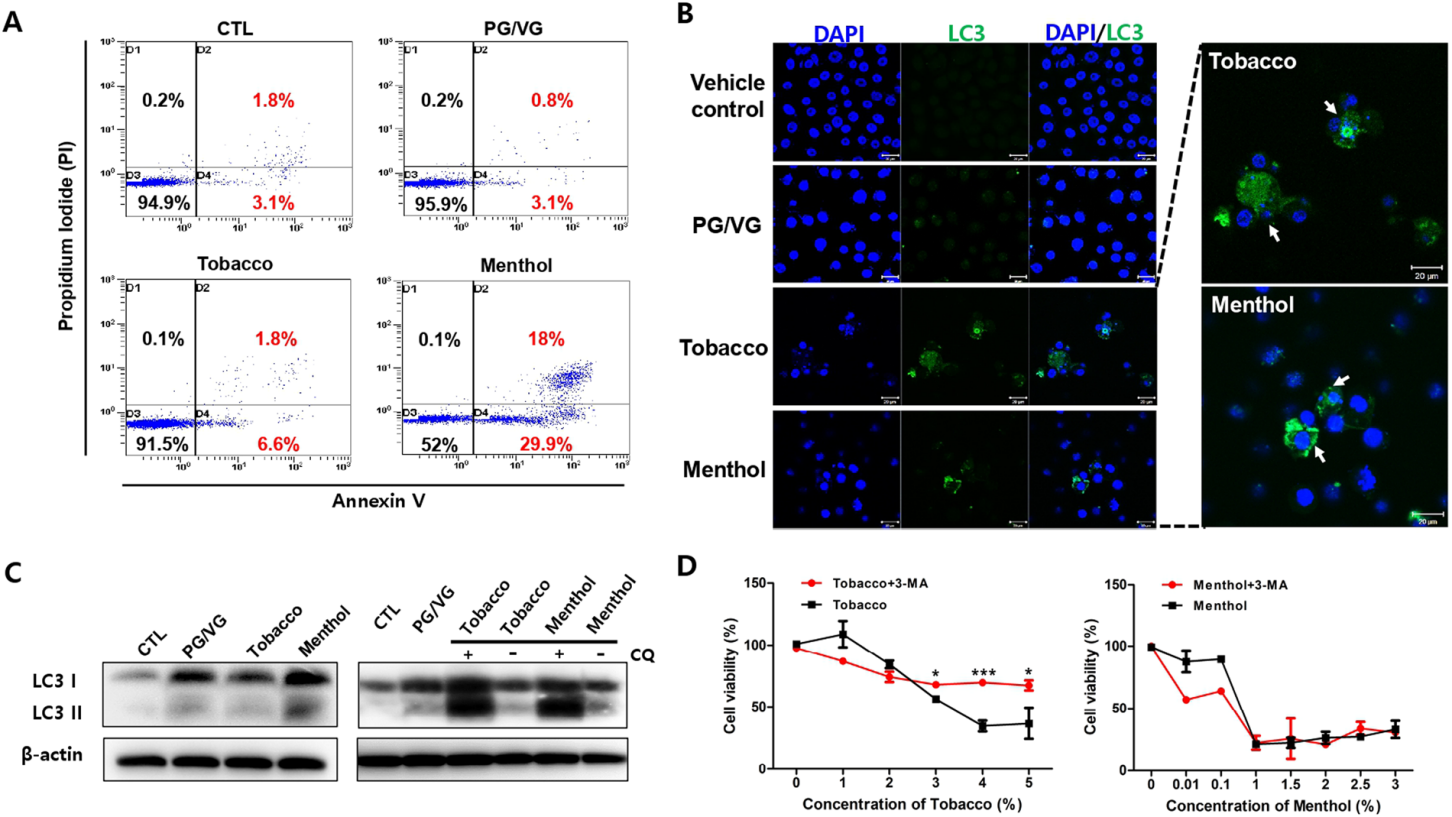
E-liquids induced apoptosis signaling in HMEECs. (**A**) HMEECs were exposed to e-liquids at the IC_50_ concentration for 24 h (PG/VG: 4.5%, Tobacco: 3.3%, and Menthol: 1.5%), and FACS was used to analyze apoptotic cells stained by annexin V-FITC/PI (blue dots). Apoptotic- and necrotic-positive cells (positive staining) were higher in the group treated with the menthol-flavored e-liquid than in the other groups. (**B**) Representative confocal microscopic images of LC3 expression in HMEECs after treatment with e-liquids (PG/VG: 4.5%, Tobacco: 3.3%, and Menthol: 1.5%) or without treatment for 24 h. LC3 expression (green) in cells was higher in e-liquid-treated groups than in the control group. Nuclei were counterstained with DAPI (blue). (**C**) Western blotting analysis of LC3 expression in HMEECs treated with PG/VG (4.5%), tobacco- (3.3%) and menthol- (1.5%) flavored e-liquids in the absence or presence of 10 μM CQ for 1 h. Cells were pre-treated with CQ for 1 h. LC3 II was significantly accumulated in cells treated with tobacco- and menthol-flavored e-liquids. Equal protein loading was verified and normalized to β-actin levels. (**D**) HMEECs were pre-treated with 3-MA (5 mM) for 1 h, and then, tobacco- and menthol-flavored e-liquids (Tobacco: 3.3%, and Menthol: 1.5%) were added at the indicated concentrations for 24 h (0.01 to 5%). Cell viability assay was performed by CCK8 method as described in Materials and Methods section. More prominent inhibition of autophagy restored cell viability of tobacco-flavored e-liquid treated group compared with menthol-flavored e-liquid. All data were obtained from three independent experiments and the error bars indicate the mean ± SD. **p < 0.01 and ***p < 0.001 compared to the corresponding control.

The previous studies suggested that apoptosis and autophagy induction are the critical response of middle ear epithelium during OM^22,23^. To investigate the mechanism of cell death when the cells were exposed to e-liquids, we first analyzed apoptosis signaling by flow cytometry analysis. After treatment with e-liquids for 24 h, the percentage of apoptotic and necrotic cells stained with annexin V-FITC/PI was determined. We observed the populations of apoptotic and necrotic-positive cells at the PG/VG (3.9%) and tobacco-flavored (8.4%) e-liquid treated groups. In particular, apoptotic and necrotic-positive staining in menthol-flavored e-liquid (47.9%) was higher than that in tobacco-flavored e-liquid treated group (Fig. 5A). Besides, the higher expression levels of Bax & caspase 3 genes and the lower expression levels of anti-apoptotic marker gene such as Bcl2 were determined in both tobacco- and menthol-flavored e-liquid compared with control group (Fig. S2). Next, we also planned to determine whether autophagy is involved in tobacco-flavored e-liquid induced cell death, since autophagy exhibits type II programmed cell death when autophagic flux is excessive in cells. To evaluate whether autophagy is induced by e-liquids in HMEECs, we first analyzed the expression of endogenous LC3 (light chain 3) in HMEECs by immunofluorescence. We detected increased accumulation of LC3, a sign of autophagy, in only flavored e-liquid-exposed HMEECs compared to the diffuse pattern in control cells (Fig. 5B). In addition, we evaluated alterations in the levels of LCI to LC3II proteins by immunoblotting, which reflects the formation of autophagosomes. Treatment with tobacco-flavored e-liquid induced a slight increase in LC3II level in HMEECs compared to that in control group. To elucidate the autophagic flux induced by tobacco-flavored e-liquid, we added the late-phase autophagy inhibitor, chloroquine (CQ). CQ caused high accumulation of LC3II in cells treated with both tobacco-flavored and menthol-flavored e-liquids compared to that in control cells (Fig. 5C). This result indicated that both flavored e-liquids induced autophagy in HMEECs. Furthermore, inhibition of autophagy by 3-methyladenine (3-MA) significantly relieved the tobacco-flavored e-liquid mediated cytotoxicity (Fig. 5D). However, menthol-flavored e-liquid-exposed HMEECs did not change the cell viability in the presence or absence of 3-MA. Taken together, these results showed that flavored e-liquids caused cell death by inducing apoptosis and autophagy in HMEECs.

## Discussion

Previous studies suggested that E-cigarettes may induce various human diseases, such as lung and bladder carcinoma, and cardiovascular harm^8,9^. However, the correlation between the cytotoxic effects of e-cigarettes and human middle ear diseases is unclear. Previously, we investigated whether environmental factors, such as Asian sand dust, urban particles, diesel particles, and cigarette smoking, cause OM [2, 4–6]. In this study, we demonstrated that e-liquids, especially flavored e-liquids, are cytotoxic and cause OM in middle ear epithelial cells by reducing cell viability and stimulating inflammatory cytokines and mucin production.

Many studies have reported that inflammatory cytokines contribute to the development of OM^24,25^. COX-2 catalyzes the synthesis of prostaglandins (PGs) and induces inflammation responses. TNF-α is a well-known cytokine and cell signaling factor involved in systemic inflammation and the acute phase reaction. It induces an inflammatory response followed by increase of mucin production in the middle ear^26^. In this study, we demonstrated that e-liquids of e-cigarettes induce inflammatory responses by activating COX-2 and TNF-α in HMEECs.

Among the mucin family members, the expressions of *MUC1*, *MUC2*, *MUC4*, *MUC5AC*, and *MUC5B* have been detected in the human nasal polyp and middle ear epithelial cells^18,27^. MUC5B is associated with mucociliary clearance and is required for defense against infections of the airway and middle ear. MUC5B has been specifically identified in human middle ears affected by COM [19, 20]. As shown in Fig. 3A, the mRNA level of *MUC5B* was higher in the PG/VG-treated group compared to the other groups. This result indicated that the immune system in middle ear epithelial cells is significantly activated by exposure to PG/VG agents. Only the basic solvents of e-liquid can induce mucin production associated with COM.

We also observed dysregulated expression levels of water channel-related genes. Since, water transport is essential for maintaining osmotic pressure in the middle ear mucosa, various water channels are vital in the progression of OM. ENaC, the epithelial sodium channel, is placed on the membrane of epithelial cells that selectively controls the permeability of Na^+^ ions^20^. It is major regulator for maintaining sodium concentration and water homeostasis by controlling active Na^+^ ions reabsorption. In addition, an OM disease model showed decreased ENaC expression in previous studies^21^. Our study also demonstrated that *ENaC* mRNA levels were significantly decreased after treatment with e-liquids, indicating that fluid accumulation in the middle ear cavity can be caused by flavored or non-flavored e-liquids via ENaC inactivation.

In this study, tobacco- and menthol-flavored e-liquids stimulated LC3 expression on HMEECs (Fig. 5B,C), reflecting increased autophagosome production after treatment with flavored e-liquids. However, it was difficult to detect the increased LC3 II/LC I ratio after treatment with e-liquids for 24 h. Therefore, HMEECs were pre-treated with CQ, the late stage of autophagy inhibitor, and we examined the increase in LC3 II levels in both tobacco- and menthol-flavored e-liquid-treated cells. These results indicated that flavored e-liquids could promote autophagosome production at the early stage of autophagy, but not inhibition of autophagosome degradation during late stage autophagy. Interestingly, cell viability was significantly improved when HMEECs were treated with the high concentration of tobacco-flavored e-liquid in the presence of 3-MA (Fig. 5D). This result indicated that tobacco-flavored e-liquid-induced cell death is mainly dependent on autophagy process. It also implied that the low concentration of tobacco-flavored e-liquid (>2.5%) induced protective autophagy, but, high concentration of tobacco-flavored e-liquid (<2.5%) induced a cell death-mediated autophagy in HMEECs.

The study from Sobolewski *et al*. supported our finding that COX-2 exhibits anti-apoptotic effects and serves as a link between apoptosis and autophagy^28^. In our results, tobacco-flavored e-liquid strongly induced COX-2 expression, although the cytotoxicity of tobacco-flavored e-liquid was lower than that of menthol-flavored e-liquid. These results indicated that tobacco-flavored e-liquid might promote COX-2-induced autophagic pathway and anti-apoptosis for cell survival of HMEECs (Fig. 5).

In the present study, we evaluated the toxicity of e-cigarettes in an *in vitro* model using immortalized human middle ear epithelial cells. However, e-cigarettes users inhale e-liquid aerosols rather than e-liquids. Therefore, further studies are needed to evaluate the effects of e-liquid aerosols using an appropriate inhalation system and an *in vivo* model.

**Figure 6:**
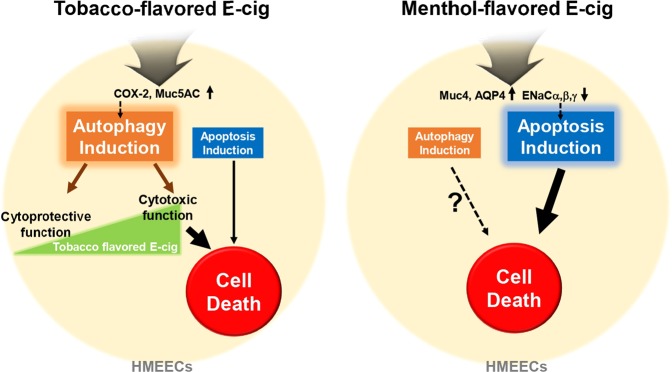
Schematic representation of the proposed cytotoxic mechanisms underlying the flavor-dependent effects of E-liquids on HMEECs.

In conclusion, our study suggested the differential mechanism of cytotoxicity between menthol and tobacco-flavored e-liquids on HMEECs due to stimulated inflammatory responses, mucin production, and dysregulation of water channels in cell membrane. Tobacco flavored e-liquid significantly induced COX-2 and Muc5AC expression, which mainly served as a possible mechanism in autophagic cell death. In contrast, menthol-flavored e-liquid exhibited apoptotic cell death on HMEECs by inducing transcription of Muc4 and AQP4 and inactivating ENaC family members (Fig. 6). Our present study could not verify the exact mechanism between OM-related cellular responses and cell death pathway when flavor containing e-liquids were exposure on HMEECs. However, these findings showed that flavor containing e-cigarettes can cause apoptotic and autophagic cell death in middle ear epithelium by inducing inflammatory responses and mucin production, thus acting as a risk factor for OM incidence.

## Materials and Methods

### Preparation of e-liquids

Tobacco and menthol-flavored e-cigarettes containing PG- and VG-based e-liquids and 9.9 mg/mL of nicotine were evaluated. These two e-liquids were selected based on their toxicity in HMEECs, and the other types of flavors, included were tobacco, menthol, coffee, fruit, and mint^16^. E-liquids were purchased from local retailers in Seoul, South Korea. Pure PG and VG were purchased from Korea Bio Medical (Seoul, Korea) and used as a control e-liquid without flavor or nicotine.

### Cell culture

HMEECs immortalized with the *E6/E7 gene* of human papilloma virus type^29^ were cultured in a mixture of Dulbecco’s modified Eagle’s medium (Lonza, Basel, Switzerland) and bronchial epithelial basal medium (Lonza) (1:1) supplemented with 10% foetal bovine serum (FBS) and 1% penicillin/streptomycin. The cells were cultured at 37 °C in a humidified atmosphere with 5% CO_2_ in a controlled incubator.

### Cell counting assay (CCK-8)

The cytotoxicity of e-liquids and cell viability were measured by the Cell Counting Assay (CCK-8 Assay, Dojindo Laboratories, Kumamoto, Japan). HMEECs (1 × 10^4^) were cultured in 96-well culture plates for 24 h. When the cells reached 80% confluence, the culture medium was replaced with serum-free fresh medium 2 h prior to treatment. The cells were treated with different concentrations of e-liquids (0, 0.01, 0.1, 1, 2, 3, 4, and 5%) for 24 h. The control group was not exposed to e-liquids. Cell viability was analyzed by the CCK-8 assay method according to the manufacturer’s instructions. After 24 h of exposure to e-liquids, 10% CCK-8 reagent was added, and the plates were incubated on a shaker for 45 min at room temperature. The optical density was measured at 450 nm using a microplate reader (Spectra Max plus 384; Molecular Devices, Sunnyvale, CA, USA). ED_50_ plus v 1.0 software was used to calculate the IC_50_ (half maximal inhibitory concentration) of the e-liquids as previously described^30^.

### RNA isolation and quantitative real-time RT-PCR

Total RNA from e-liquid-treated and -untreated HMEECs was isolated using Trizol reagent (Invitrogen, Carlsbad, CA, USA), according to the manufacturer’s instructions. For quantitative real-time PCR analysis, 1 µg of RNA was reverse-transcribed using a cDNA synthesis kit (Bioneer, Inc., Daejeon, Korea). Quantitative real-time PCR was performed using the Quant Studio 6 Flex Realtime PCR system (Thermo Fisher Scientific, Waltham, MA, USA) and 50 ng of cDNA. The real-time PCR mixture contained Power SYBR^TM^ Green PCR Master Mix (Life Technologies, Carlsbad, CA, USA), and 1 µL of 5 pmol forward and revers primers in a total reaction volume of 20 µL. Real-time PCR was performed by denaturation at 95 °C for 15 s and annealing at 60 °C for 1 min for 40–45 cycles. All reactions were performed in triplicates. The target mRNA expression level was normalized to that of GAPDH and Ct was calculated using the comparative Ct method. The specific primers of genes used for quantitative real-time PCR are listed in the supplemental experimental procedure.

### Western blot analysis

For western blot analysis, HMEECs were cultured in 6-well culture plates with 5 × 10^5^ cells in each well. At 80% confluence, the cells were starved in serum-free growth medium for 2 h. The cells were exposed to e-liquids at the IC_50_ concentration and further incubated for 24 h. After incubation, the medium was removed, and the cells were washed twice with PBS (phosphate-buffered saline; 10 mM, pH 7.4). Lysis buffer (0.1 µL of 50 mM Tris-HCl (pH 7.4,) 150 mM NaCl, 1% Triton X-100, 0.5% sodium deoxylcholate, 0.1% SDS, 1 mM EDTA) containing protease inhibitor cocktail (Roche Diagnostics, Indianapolis, IN, USA) and phosphatase inhibitor (Roche Diagnostics) was added to the cells, vortexed, and incubated for 1 h on ice. The cells were centrifuged at 13,000 rpm for 30 min at 4 °C and the supernatant was collected. The protein concentration in the cell lysates was measured using the Quick Star Bradford dye reagent (Bio-Rad, Hercules, CA, USA). Equal quantities of proteins were separated by 12% sodium dodecyl sulphate polyacrylamide gel electrophoresis. The proteins in the gel were transferred to a polyvinyl difluoride membrane (Bioneer, Inc.) and the membranes were blocked with TBST (494 mM Tris, 2.74 M NaCl, 54 mM KCl, pH 7.4 using HCl) containing 5% (w/v) skim milk for 30 min at room temperature. Membranes were probed with primary antibodies against cyclooxygenase 2 (COX-2) (1:200; Santa Cruz Biotechnology, Dallas, TX, USA), tumor necrosis factor-α (TNF-α) (1:200; Santa Cruz Biotechnology), and β-actin (1:2,000; Santa Cruz Biotechnology). The following day, membranes were washed with TBST and incubated with a secondary antibody against horseradish peroxidase-conjugated anti-mouse IgG (Invitrogen, 1:5,000 in TBST containing 5% skim milk) for 1 h at room temperature, and then, the membranes were washed with TBST. The membranes were developed using the enhanced chemiluminescence detection solution (Dogenbio, Seoul, Korea) and signals were captured using a Fusion Solo Imaging System (Vilber Lourmat, Marne-la-Vallée, France). Full-length gels and blots in Figs. 3C and 5C are included in a supplemental experimental procedure.

### Immunofluorescence staining

HMEECs (4 × 10^4^) were seeded into a chamber slide and grown for 24 h. When the cells reached 80% confluence, they were starved for 2 h, and exposed to e-liquids at the IC_50_ concentration for 24 h. After 24 h, the cells were fixed with 4% paraformaldehyde for 15 min at room temperature and washed briefly with PBS for 10 min. After fixation, the cells were incubated for 15 min in PBS containing 0.5% Triton X-100 to permeabilize the cell membrane, and then, washed with PBS for 10 min. The cells were blocked with 3% bovine serum albumin in PBS at 4 °C for 30 min and probed with a primary antibody against MUC5AC (Invitrogen, 1:200 in 3% bovine serum albumin) at 4 °C for 24 h. The following day, the cells were washed with TBST for 15 min, incubated with secondary antibody against anti-mouse Alexa488-conjugated antibody (Invitrogen, 1: 500 in PBS) for 15 min at 4 °C in the dark, washed with TBST for 15 min, and counterstained with 4, 6-diamidino-2-phenylindole (DAPI) for nuclear staining. The cells were washed with TBST for 15 min and mounted with a drop of mounting medium (Vector Laboratories, Inc., Burlingame, CA, USA). Coverslips were sealed with nail polish to prevent drying and movement under the microscope. Finally, the fluorescence signals in the cells were analyzed by confocal microscopy (Zeiss LSM700, Oberkochen, Germany).

### Annexin V-fluorescence isothiocyanate (FITC)/propidium iodide (PI) flow cytometry

To perform Annexin V/PI flow cytometry, the cells were analyzed with an Annexin V-FITC Apoptosis Kit (SouthernBiotech, Birmingham, AL, USA) according to the manufacturer’s protocol. HMEECs were cultured in 6-well culture plates with 5 × 10^5^ cells in each well. At 80% confluence, the cells were starved in serum-free growth medium without FBS for 2 h. The cells were exposed to e-liquids at the IC_50_ concentration (determined by CCK-8 assay) and incubated for 24 h. Next, the medium and cell lysates were collected and centrifuged at 1,000 rpm for 5 min. After collection, the cells were washed with PBS, resuspended in 100 µL of 1X binding buffer, and mixed with 10 µL of conjugated Annexin V-FITC solution. The cells were gently vortexed, incubated for 15 min at 4 °C in the dark, and mixed with 1 X binding buffer (up to 500 µL for each sample) and 10 µL of propidium iodide (PI). The cells were analyzed with a flow cytometer (BD Biosciences, San Diego, CA, USA).

### Statistical analysis

The results are expressed as the mean ± standard deviation (SD). The data were analyzed using the Student’s *t*-test to determine the significance of the difference between groups. Statistical analyses were performed using GraphPad QuickCalcs software (GraphPad, Inc., La Jolla, CA, USA). A *p* value < 0.05 was considered as significant.

## Supplementary information

Supplementary Information.
