# Supplementary material for: Comparison between in vitro toxicities of tobacco- and menthol-flavored electronic cigarette liquids on human middle ear epithelial cells
Source: Sci Rep. 2020 Feb 13;10:2544. doi: 10.1038/s41598-020-59290-y (PMC7018737; doi:10.1038/s41598-020-59290-y)
Supplement: Supplementary file 1 — Supplementary Information. [file 41598_2020_59290_MOESM1_ESM.pdf]

Comparison between *in vitro* toxicities of tobacco- and menthol-flavored electronic cigarette liquids on human middle ear epithelial cells

Yoon Young Go[1], Ji Yoen Mun[1], Sung-Won Chae[1], Jiwon Chang[2]*, and Jae-Jun Song[1]*

Supplementary figures and figure legends

Figure. S1. Time-dependent cytotoxicity of tobacco- and menthol-flavored e-liquid on HMEECs.

HMEECs were treated with PG/VG, tobacco- and menthol-flavored e-liquids for 1, 6, 16, and 24 h in a various concentration of e-liquid (1.5 to 10%). The control group was not exposed to e-liquids. CCK8 assay used to determine cytotoxicity on HMEECs. Cell viability reduced by exposure to e-liquids in a time and concentration-dependent manner. Experiments were performed in triplicate. $^{**}p < 0.01$ compared to the untreated control group.

Figure S2. mRNA expression of apoptosis-related marker genes in E-liquid treated HMEECs.

HMEECs were treated to e-liquids at the IC_{50} concentration for 24 h (PG/VG: 4.5%, Tobacco: 3.3%, and Menthol: 1.5%), and quantitative RT-PCR was performed to investigate the expression levels of apoptosis-related genes, *Bax*, *Bcl2*, and *caspase 3*. The apoptosis marker such as *Bax* and *caspase 3* genes were significantly increased in e-liquid treated HMEECs. Conversely, the mRNA levels of *Bcl2* were notably decreased in both e-liquid treated groups compared with control. All data were obtained from three independent experiments and the error bars indicate the mean ± SD. $^{*}p < 0.05$, $^{**}p < 0.01$, and $^{***}p < 0.001$ compared to the corresponding control.

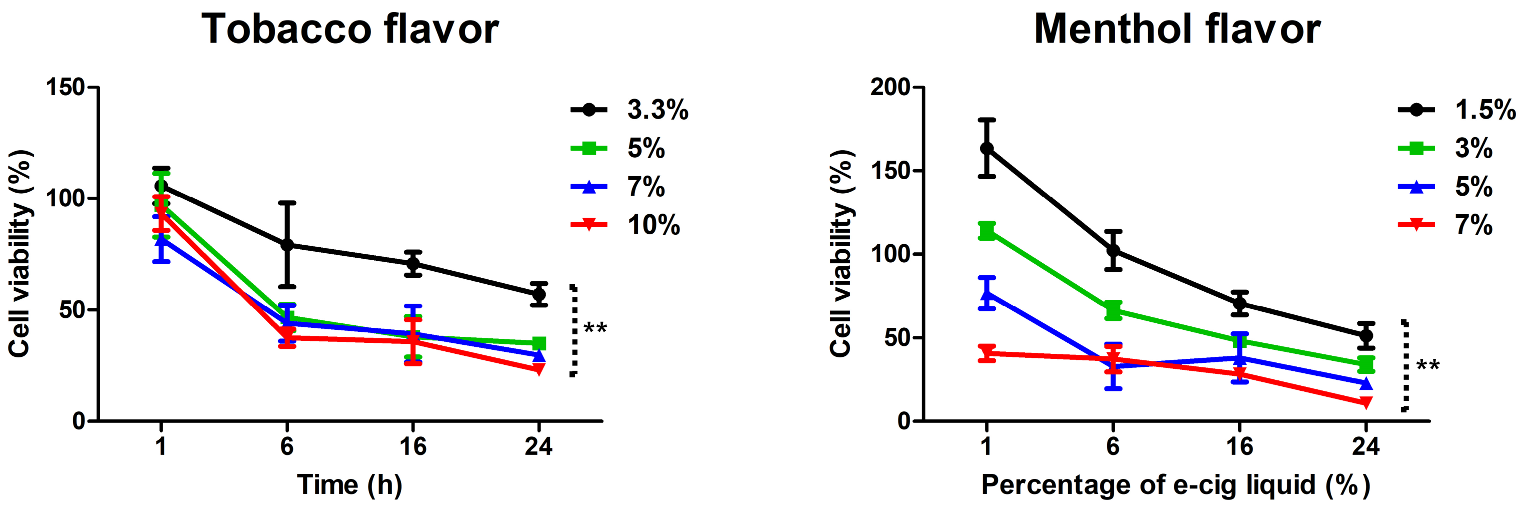

Tobacco flavor
Cell viability (%)
150
100
50
0
1
6
16
24
Time (h)
3.3%
5%
7%
10%
**
Menthol flavor
Cell viability (%)
200
150
100
50
0
1
6
16
24
Percentage of e-cig liquid (%)
1.5%
3%
5%
7%
**

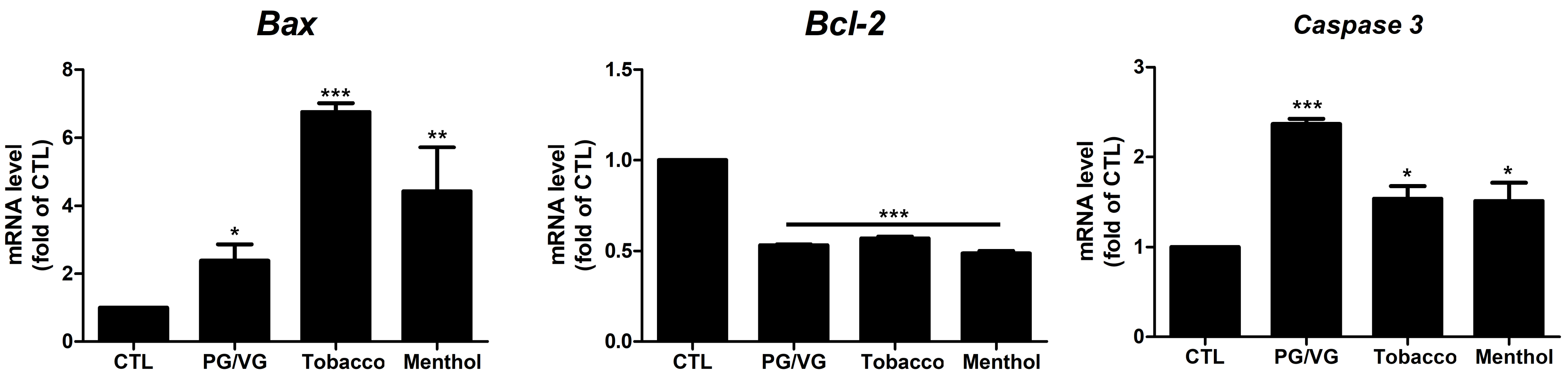

Bax
8
6
4
2
0
mRNA level
(fold of CTL)
*
***
**
CTL
PG/VG
Tobacco
Menthol
Bcl-2
1.5
1.0
0.5
0.0
mRNA level
(fold of CTL)
***
CTL
PG/VG
Tobacco
Menthol
Caspase 3
3
2
1
0
mRNA level
(fold of CTL)
***
*
*
CTL
PG/VG
Tobacco
Menthol

Supplemental Experimental Procedures

Table 1. Sequences of oligonucleotide primers for quantitative RT-PCR.

Primer	Direction	Sequence
COX-2	forward	5'-CTGGCGCTCAGCCATACAG-3'
	reverse	5'-CGCACTTATACTGGTCAAATCCC-3'
TNF-α	forward	5'-GAGGCCAAGCCCTGGTATG-3'
	reverse	5'-CGGGCCGATTGATCTCAGC-3'
MUC5AC	forward	5'-ACCCTGCTCCTGGAATAAATG -3'
	reverse	5'-CTGGTTCTTGTTCAGGCAAATC-3'
MUC5B	forward	5'-GCCTACGAGGACTTCAACGT-3'
	reverse	5'-CCTTGATGACAACACGGGTG -3'
MUC4	forward	5’-CTTACTCTGGCCAACTCTGTAGTG-3’
	reverse	5’-GAGAAGTTGGGCTTGACTGTC-3’
ENaC-α	forward	5’-GCAGTCCGATTTGTTCTGGT-3’
	reverse	5’-CAGGTGGACTGGAAGGACTG-3’
ENaC-β	forward	5’-GACCAAAGCACCAATATCACC-3’
	reverse	5’-GAAGTAGATGTTGAGCTTGACAATTC -3’
ENaC-γ	forward	5’-TCTACAACGCTGCCTACTCG -3’
	reverse	5’-TCCACCATCTTTGTCTGGAA -3’
AQP4	forward	5’-TCTCATCTCCCTTTGCTTTG -3’
	reverse	5’-ACAGTCACTGCAGGGTTGAT -3’
GAPDH	forward	5'-TCGCCCCACTTGATTTTGG-3'
	reverse	5'-GCAAATTCCATGGCACCGT-3'

Full-length blots in Figure 3C

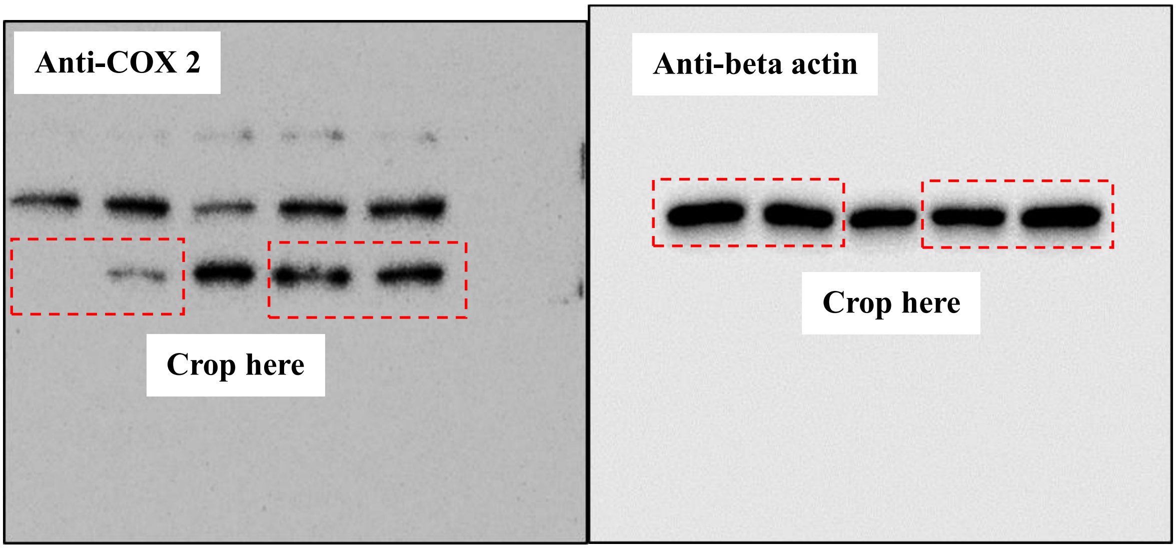

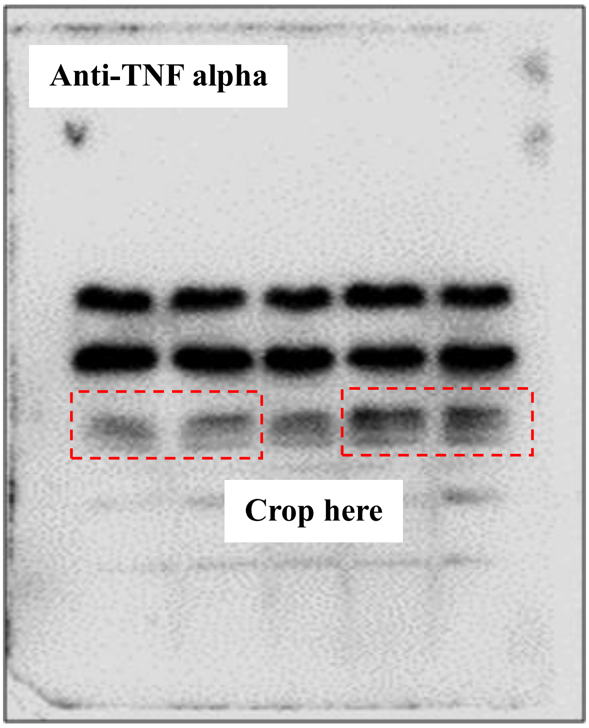

Full-length blots in Figure 5C

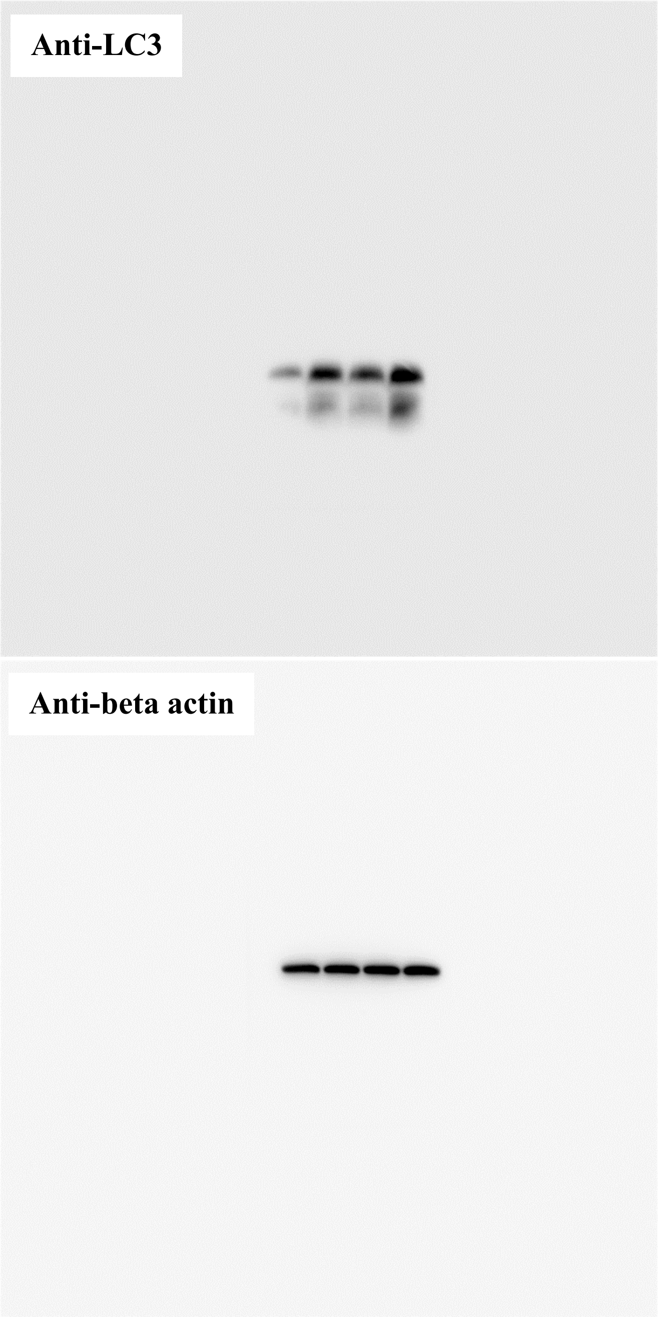

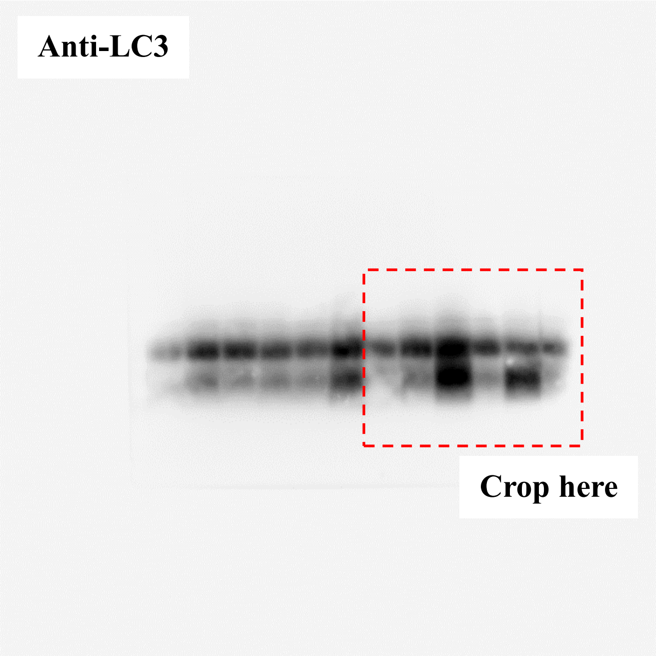
Anti-LC3
Crop here

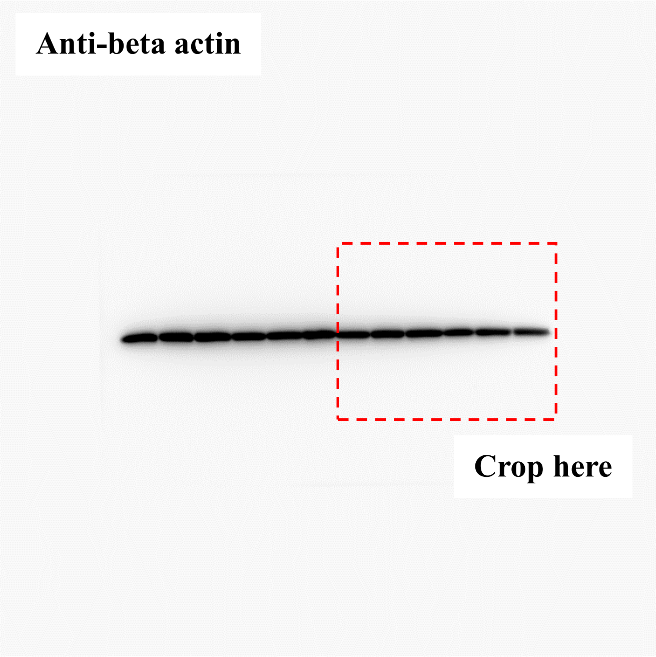
Anti-beta actin
Crop here
